# Role of Vitamin D Deficiency in the Pathogenesis of Cardiovascular and Cerebrovascular Diseases

**DOI:** 10.3390/nu15020334

**Published:** 2023-01-09

**Authors:** Éva Pál, Zoltán Ungvári, Zoltán Benyó, Szabolcs Várbíró

**Affiliations:** 1 Institute of Translational Medicine, Semmelweis University, 1094 Budapest, Hungary; 2 Eötvös Loránd Research Network, Semmelweis University (ELKH-SU), Cerebrovascular and Neurocognitive Disorders Research Group, 1094 Budapest, Hungary; 3 Vascular Cognitive Impairment and Neurodegeneration Program, Oklahoma Center for Geroscience and Healthy Brain Aging, Department of Biochemistry and Molecular Biology, University of Oklahoma Health Sciences Center, Oklahoma City, OK 73104, USA; 4 Department of Health Promotion Sciences, College of Public Health, University of Oklahoma Health Sciences Center, Oklahoma City, OK 73104, USA; 5 International Training Program in Geroscience, Doctoral School of Basic and Translational Medicine/Departments of Public Health and Translational Medicine, Semmelweis University, 1085 Budapest, Hungary; 6 The Peggy and Charles Stephenson Cancer Center, University of Oklahoma Health Sciences Center, Oklahoma City, OK 73104, USA; 7 Department of Obstetrics and Gynecology, Semmelweis University, 1082 Budapest, Hungary; 8 Workgroup for Science Management, Doctoral School, Semmelweis University, 1085 Budapest, Hungary

**Keywords:** vitamin D, cholecalciferol, vitamin D2, ergocalciferol, aging, cerebral circulation, vascular cognitive impairment, VCI, stroke, cerebrovascular disease, hypertension

## Abstract

Deficiency in vitamin D (VitD), a lipid-soluble vitamin and steroid hormone, affects approximately 24% to 40% of the population of the Western world. In addition to its well-documented effects on the musculoskeletal system, VitD also contributes importantly to the promotion and preservation of cardiovascular health via modulating the immune and inflammatory functions and regulating cell proliferation and migration, endothelial function, renin expression, and extracellular matrix homeostasis. This brief overview focuses on the cardiovascular and cerebrovascular effects of VitD and the cellular, molecular, and functional changes that occur in the circulatory system in VitD deficiency (VDD). It explores the links among VDD and adverse vascular remodeling, endothelial dysfunction, vascular inflammation, and increased risk for cardiovascular and cerebrovascular diseases. Improved understanding of the complex role of VDD in the pathogenesis of atherosclerotic cardiovascular diseases, stroke, and vascular cognitive impairment is crucial for all cardiologists, dietitians, and geriatricians, as VDD presents an easy target for intervention.

## 1. Introduction

Vitamin D (VitD) is a lipid-soluble vitamin that functions as a steroid hormone [1]. VitD is best known for its role in calcium and bone homeostasis. Since the public has been educated about the importance of VitD, foods are now fortified with VitD and VitD containing dietary supplements are widely available, public health authorities thought that health problems resulting from VitD deficiency (VDD) had been resolved. Yet, it became apparent that, while rickets due to severe VitD is indeed very rare, moderate VDD is still quite prevalent and represents an important health challenge.

Estimates of the prevalence of VDD ranges from 24% (United States) to 40% (European Union) [2,3,4,5,6,7,8]. It is now evident that, in addition to the musculoskeletal system, most tissues and cell types express VitD receptors and VitD, in addition to its role in promoting and maintaining skeletal health, confers complex health benefits in multiple organ systems, including the immune system and the cardiovascular system [9,10,11]. Accordingly, efforts have been made to examine the causes, complex consequences, and prevention strategies of the “world pandemic” of VDD [2]. VDD has already been linked to increased risk for several diseases, including common cancers (colon, breast, and prostate cancers), diabetes mellitus, coronary artery disease, ischemic stroke, and autoimmune diseases [2].

Cardiovascular and cerebrovascular diseases belong to the leading causes of death and disability worldwide [12,13]. Importantly, the prevalence of cardiovascular diseases has been reported to increase with aging [14]. Since the average life expectancy is increasing globally, the burden to prevent and treat cardiovascular diseases will increase in the following decades. Several studies investigating the physiological and pathophysiological properties of the cardiovascular and cerebrovascular system have focused on the role of endothelial function in the maintenance of vascular health. Endothelial dysfunction, characterized by imbalanced vasodilation and vasoconstriction, elevated reactive oxygen species, and proinflammatory factors, decreases nitric oxide bioavailability and contributes significantly to the development and progression of cardiovascular and cerebrovascular diseases [15,16,17]. Although many mechanisms and risk factors of cardiovascular disorders have already been identified, the prevention and treatment strategies of vascular diseases are yet to be improved. To preserve cardiovascular health, even in advanced age, the pathogenesis and the risk factors of cardiovascular diseases have to be further investigated. In addition to traditional risk factors (e.g., hypertension, diabetes mellitus), VDD appears to increase the risk of cardiovascular and cerebrovascular disorders [1,13,18].

In this review, the effects of VitD on the functional and structural integrity of the circulatory system are considered, in terms of potential mechanisms involved in endothelial dysfunction and accelerated vascular aging phenotypes associated with VDD. The role of VDD in the pathogenesis of specific cardiovascular and cerebrovascular diseases (including stroke and vascular cognitive impairment (VCI)) is discussed [13].

## 2. Vitamin D Biosynthesis and Metabolism

Vitamin D can represent either vitamin D_2_ (ergocalciferol) or vitamin D_3_ (cholecalciferol), both of which are produced naturally by ultraviolet B radiation (290 to 315 nm wavelength) from ergosterol in yeast and mushrooms or from 7-dehydrocholesterol in the epidermis [2]. Humans acquire VitD mainly from its precursors upon exposure to sunlight and, to a lesser extent, from certain foods, such as oily fish [1]. Following the exposure of skin to sunlight, 7-dehydrocholesterol is converted first to pre-vitamin D_3_, which spontaneously isomerizes to vitamin D_3_ in a thermosensitive process [19]. Vitamin D_2_ or D_3_ from ingested food is incorporated into chylomicrons, followed by absorption into the lymphatic system and entering the venous blood. Inactive vitamin D (as well as its metabolites) circulates within the blood stream bound to carrier proteins, mainly to the VitD binding protein [20], and is subsequently metabolized in two steps to its active form, 1,25-dihydroxyvitamin D (1,25(OH)_2_D, calcitriol). First, the biomarker of VitD status, 25-hydroxyvitamin D (25(OH)D) is produced mostly by cytochrome P450 (CYP) 2R1 (but also by CYP27A1, CYP3A4, and CYP2J3) in the liver [2]. Next, 1,25(OH)_2_D is formed exclusively by CYP27B1 (25-hydroxyvitamin D-1α-hydroxylase), particularly in the kidney [19,21]. In addition to the kidney, many extrarenal tissues express CYP27B1; therefore, those are also capable of producing the active form of VitD [22]. The extrarenal production of 1,25(OH)_2_D is stimulated mainly by cytokines and appears to be important in the paracrine regulation of cell function [23]. Unlike extrarenal CYP27B1, renal 1α-hydroxylase is tightly regulated by the parathyroid hormone, fibroblast growth factor 23, and the plasma levels of 1,25(OH)_2_D, calcium, and phosphate ions [19,22]. In order to avoid the accumulation of 1,25(OH)_2_D or 25(OH)D, the target cells of VitD express CYP24A1 (24-hydroxylase), which converts 1,25(OH)_2_D to biologically inactive calcitroic acid [19], whereas, in the kidney, it is 24-hydroxylase catabolyses 25(OH)D, when a sufficient amount of 1,25(OH)_2_D has already been produced [21].

## 3. Mechanism of Action of Vitamin D

### 3.1. Genomic Actions

**Figure 1:**
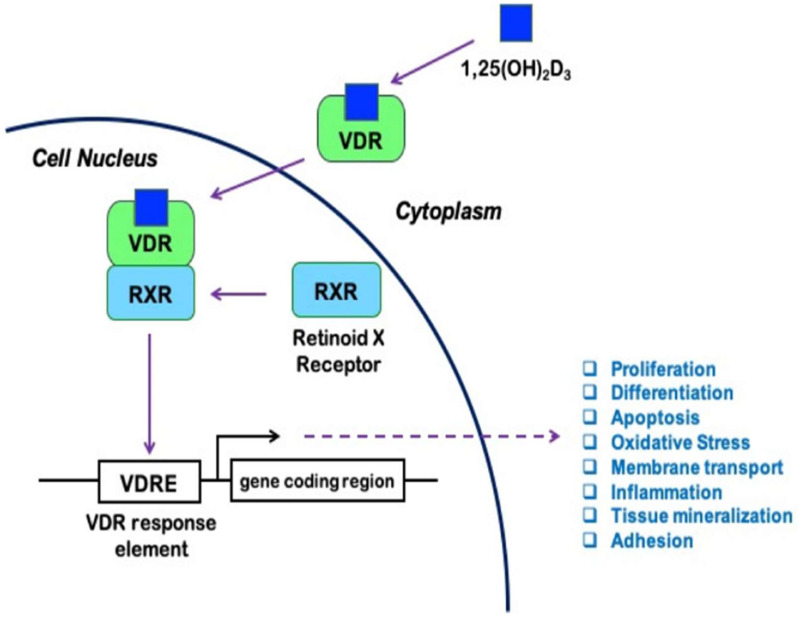
Genomic actions of vitamin D. 1,25-dihydroxyvitamin D_3_ (1,25(OH)_2_D_3_) binds to the vitamin D receptor (VDR) and promotes its heterodimerization with the retinoid X receptor (RXR). The ligand-bound VDR/RXR complex binds to the vitamin D response elements (VDRE) in the promoters of numerous genes and modulates their transcription. Therefore, 1,25(OH)_2_D_3_ regulates several physiological processes, such as cell proliferation, differentiation, and inflammation [13]. Figure reproduced from “Vitamin D Deficiency and the Risk of Cerebrovascular Disease.” by Kim et al., Antioxidants (Basel), 2020, 9, 327, doi:10.3390/antiox9040327. The original figure was published (and can be reproduced) under the terms of CC-BY 4.0 [13].

The biological actions of 1,25(OH)_2_D are mediated by the vitamin D receptor (VDR), which belongs to the nuclear receptor superfamily and acts as a ligand-activated transcription factor [20]. VDR was first discovered in chicken intestines [24], but was later found to be present in almost all cells and tissues [25]. VDR regulates the expression of numerous genes the promoters that contain specific DNA sequences known as vitamin D response elements (VDRE) [1,25]. The binding of 1,25(OH)_2_D to VDR induces conformational changes in the receptor that facilitate its interaction with the retinoid X receptor (RXR) and, subsequently, the formation of a VDR/RXR heterodimer, which provides adequate DNA binding affinity [20,26]. The ligand-bound VDR/RXR heterodimeric complex binds to the VDRE on the target genes and acts as a transcription factor that up- or downregulates their transcription [20,27]. The action of 1,25(OH)_2_D depends, however, on the involvement of tissue specific co-factors, for instance, the steroid-specific coactivators, and subsequently on the formation of transcriptional complexes [25]. The genomic actions of 1,25(OH)_2_D are schematically shown in Figure 1.

### 3.2. Non-Genomic Actions

Interestingly, VitD has been reported to have some rapid actions that are unlikely to involve the direct regulation of gene expression. These effects may, rather, be mediated by a membrane-associated VDR, which has been less well-characterized than the nuclear VDR [25]. The non-genomic actions of VitD include the activation of signaling molecules (e.g., phospholipase C (PLC), phospholipase A_2_ (PLA_2_), phosphatidylinositol-3-kinase (PI3K)), the rapid generation of second messengers, such as Ca^2+^, the activation of protein kinases, and the opening of the Ca^2+^ and Cl^−^ channels [28]. Surprisingly, however, these rapid, non-genomic actions appear to require the presence of the nuclear VDR, implying cooperation between the membrane-associated and nuclear VDRs [25]. In addition, the ligand-bound nuclear VDR has been reported to have non-classical, non-genomic actions. In that case, VitD regulates the target gene expression via protein–protein interactions, instead of binding to the VDRE on target genes [28].

## 4. Physiological Significance of Optimal Vitamin D Status

### 4.1. Determinants of Vitamin D Status

Vitamin D deficiency and insufficiency—defined usually as 25(OH)D levels below 20 ng/mL (50 nmol/L) and within the range of 21–29 ng/mL (52.5–72.5 nmol/L), respectively [10]—affect approximately one billion people worldwide [2]. Lifestyle and environmental factors limiting sunlight exposure of the skin are the main causes of VDD, but the decreased synthesis of 25(OH)D or 1,25(OH)_2_D, and heritable disorders such as hereditary VitD-resistant rickets could also reduce the bioavailability of VitD [2]. Several diseases, including cardiovascular disorders, have been associated with decreased VitD serum levels [2]. For instance, lower 25(OH)D serum levels (~27 ng/mL) have been reported in patients with hypertension, compared to normotensive individuals (~31 ng/mL) [29]. Similarly, Melamed et al. reported lower 25(OH)D levels among patients with peripheral arterial disease (21.5 ng/mL vs. 24.6 ng/mL in healthy subjects) [30]. Since there are few foods naturally containing VitD (such as cod liver oil, shiitake mushrooms, egg yolk), in general, sufficient VitD supply can be provided only by exposure to sunlight or by taking VitD supplements [2,11]. As a variety of factors could reduce the cutaneous production of VitD, such as ultraviolet protection, increased skin pigmentation, age, and seasonal and geographical variation [11], it is recommended to take VitD supplements: in general, 1000–2000 IU/day are needed to reach and maintain 25(OH)D levels greater than 30 ng/mL in the majority of the healthy population, in order to prevent VDD [10]. Unlike VDD, VitD intoxication (25(OH)D levels higher than 150 ng/mL (374 nmol/L)) is extremely rare, particularly because it cannot be caused by exposure to sunlight, since excess pre-vitamin D_3_ or vitamin D_3_ is destroyed by sunlight itself [2].

### 4.2. Physiological Role of Vitamin D

VitD appears to control the expression of more than 200 genes, as well as several signaling molecules and second messengers, including those not typically associated with mineral homeostasis (Table 1) [26,28]. The active form of VitD regulates, for instance, cellular proliferation, differentiation, apoptosis, angiogenesis, oxidative stress, membrane transport, matrix homeostasis, cell adhesion, immune functions, insulin secretion, and renin expression (Figure 1) [2,26,28,31]; thus, VitD plays an integral physiological role in nonskeletal tissues. Consequently, in addition to its well-characterized roles in calcium and phosphate homeostasis, as well as in bone metabolism, VitD exerts beneficial effects, for instance, on glucose homeostasis, the immune response, and the cardiovascular system [2]. More severe VDD impairs the mineral and bone homeostasis characterized by rickets and growth retardation in children, as well as osteomalacia, osteoporosis, and decreased muscle strength or sarcopenia in adults and the elderly [2]. Additionally, VDD is also associated with increased risk for cancer (e.g., colon, prostate, and breast cancer), diabetes mellitus, metabolic syndrome, infections, autoimmune diseases, depression, schizophrenia, and cardiovascular diseases [2,32].

Although VitD intoxication is rare, it can be caused by taking extensively high doses of VitD supplements [2]. The clinical manifestations of VitD toxicity are related primarily to hypercalcemia, and they include confusion, depression, psychosis, gastrointestinal disorders, renal failure, and cardiovascular symptoms, such as hypertension and bradyarrhythmia [39]. Surprisingly, it appears that both low and high 25(OH)D levels are associated with increased risk of total [40] and cardiovascular mortality [16,41], implying a U-shaped association between VitD concentrations and health. Although VitD appears to have a broad therapeutic window, the latter still has to be defined, especially for preventing cardiovascular and cerebrovascular diseases [31]. Nevertheless, optimal VitD supply is considered to be a prerequisite for health in all age groups [42].

## 5. Impacts of Vitamin D on the Cardiovascular System

### 5.1. Cellular Effects of Vitamin D

**Figure 2:**
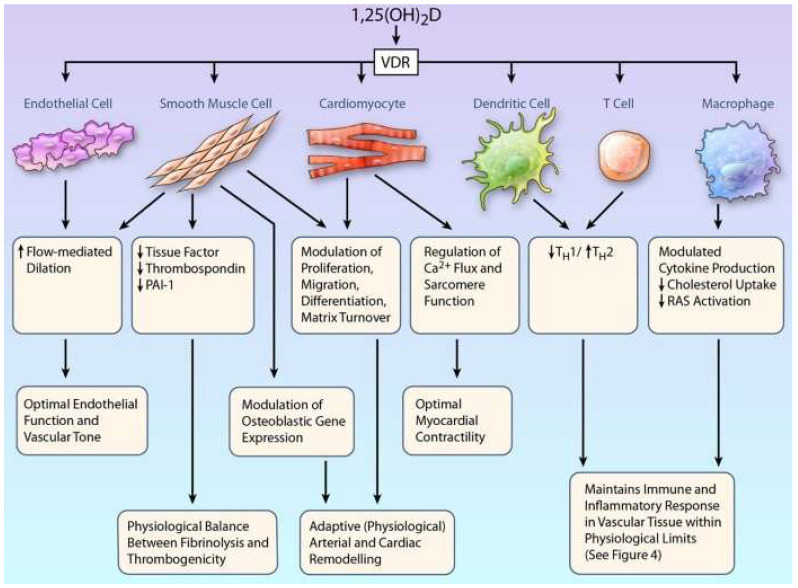
Overview of the cardiovascular system-related impacts of vitamin D (TSP: thrombospondin, PAI-1: plasminogen activator inhibitor-1, RAS: renin-angiotensin system, T_H_1: T helper type 1 cell, T_H_2: T-helper type 2 cell; “See Figure 4” refers to Figure 4 of the original article by Norman PE and Powell JT [1]). Figure reproduced from “Vitamin D and cardiovascular disease” by Norman PE and Powell JT, Circ Res 2014, 114, 379–393, doi:10.1161/circresaha.113.301241. [1] with permission of the publisher. Copyright 2014, American Heart Association, Inc.

There is a growing body of evidence linking VDD to cardiovascular diseases [1]. For instance, VDD is associated with atherosclerosis, hypertension, cardiac hypertrophy, cerebrovascular diseases, coronary heart disease, and peripheral artery disease [2], as well as with several cardiovascular risk factors, such as dyslipidemia, insulin resistance, diabetes mellitus, and abdominal obesity [1,2,32]. VitD exerts a direct effect on the cardiovascular system, since VDRs have been found in cardiomyocytes [43], vascular smooth muscle cells (VSMCs) [44], endothelial cells [45], circulating monocytes, macrophages, dendritic cells, activated T lymphocytes [46], and platelets [47]. Furthermore, CYP27B1 (25-hydroxyvitamin D-1α-hydroxylase) is expressed in most of these cells, which enables the local synthesis of 1,25(OH)_2_D [1]. The cardiovascular protective effects of VitD include the modulation of immune, inflammatory, and endothelial functions [1]. Furthermore, VitD regulates cell proliferation and migration, renin expression, and extracellular matrix homeostasis, and it may attenuate the adverse effects of advanced glycation end products on endothelial cells [1,32]. In addition, VitD has an antithrombotic effect, since it downregulates the tissue factor, plasminogen activator inhibitor-1, and thrombospondin-1, whereas it upregulates thrombomodulin expression in monocytes and VSMCs [1,48]. Furthermore, VitD inhibits formation of foam cells and cholesterol uptake by macrophages; thus, it also exerts antiatherogenic effects [32]. Figure 2 summarizes the effects of VitD related to the cardiovascular system.

### 5.2. Impacts of Vitamin D on Blood Pressure and Cardiac Functions

VitD appears to have a beneficial effect on arterial blood pressure, and consequently, VDD is linked to hypertension [1]. However, the association between VitD levels and blood pressure values is not fully confirmed [49], especially in young healthy subjects [50,51]. Nevertheless, the impact of VitD on blood pressure has been attributed particularly to the negative regulation of the renin–angiotensin system (RAS) [52], since VitD appears to decrease the activity of the cyclic adenosine monophosphate response element in the renin gene promoter [38]. Consequently, VDR deficiency increases the expression of renin and, therefore, the production of angiotensin II, which can result in hypertension and cardiac hypertrophy [52]. Surprisingly, however, normotensive VDR knockout mice also developed cardiac hypertrophy [43], which could imply that VitD acts directly on cardiomyocytes [31,53]. Accordingly, VitD has been reported to stimulate cardiomyocyte relaxation, which could improve coronary perfusion during diastole, and it also regulates the gene expression profile of the extracellular matrix in the heart [32]. In conclusion, experimental animal models of VDD indicate that VitD prevents hypertension; however, the causal association between VitD levels and blood pressure values is still not fully confirmed.

### 5.3. Effects of Vitamin D on Angiogenesis and Vascular Remodeling

VitD has been reported to regulate the expression of several genes involved in cell proliferation and differentiation [35], as well as in extracellular matrix homeostasis (Table 1) [1] and, therefore, VitD likely modulates processes of angiogenesis and vascular remodeling. Accordingly, preclinical studies show that VitD attenuates pathological vascular remodeling both in intrarenal arteries in kidney fibrosis [54] and in basilar arteries after subarachnoid hemorrhage in rodent models [55]. Further, VDD has been reported to decrease the lumen and increase the wall thickness of coronary arterioles of female rats [56]. Altered VSMC migration and proliferation may be responsible for the vascular remodeling in VDD [16]. However, the literature is controversial regarding the effect of VitD treatment on VSMCs. Some studies using rat VSMCs report enhanced migration and proliferation [57,58], whereas others found VitD-induced inhibition of rat and human VSMC growth [44,59]. The effect of VitD treatment on VSMCs appears to depend on the applied dose. For instance, physiological doses of VitD inhibit VSMC proliferation [16] via blunting c-myc RNA induction [58], up-regulating the negative modulators of cell proliferation, including transforming growth factor β (TGF-β) [33], or decreasing cyclin-dependent kinase 2 (Cdk2) activity [60].

In addition, VitD participates in the modulation of endothelial cell proliferation and matrix homeostasis, due to the regulation of vascular endothelial growth factor (VEGF) and matrix metalloproteinases (MMPs) [1]. VEGF is known to stimulate endothelial cell proliferation and migration and mediate vascular growth and angiogenesis [61], whereas MMPs regulate angiogenesis and vascular remodeling by degrading extracellular matrix proteins [62]. Experimental animal models of VDD and clinical studies have reported that VDD decreased the expression of tissue inhibitors of MMP-1 and MMP-3, but upregulated the expression of MMP-2 and MMP-9 [1]. These alterations in extracellular matrix homeostasis may contribute to the development of vascular calcification [63]. Furthermore, VitD induces the upregulation of VEGF in endothelial progenitor cells [36], as well as in mature endothelial cells [64], and in VSMCs [27,57], implying the role of VitD in vasculogenesis, angiogenesis, and endothelial repair [61]. Surprisingly, however, VitD has also been reported to inhibit angiogenesis partly via reducing the protein expression of VEGF in various human tumor cells [65]. Thus, the effect of VitD on angiogenesis is ambiguous; however, the inhibitory impact of VitD on VEGF expression and angiogenesis has been reported almost exclusively in cancer studies [66]. In addition, VitD appears to stimulate the ability of multipotent mesenchymal stromal cells to promote vasculogenesis [67].

Furthermore, VitD regulates the elastin and collagen content of the vessel wall [37]; thus, it influences vascular resistance and arterial stiffness [68]. For instance, Andrukhova et al. reported increased collagen and decreased elastin content of the ascending aorta of 9-month-old VDR-deficient mice [37]. Correspondingly, Salum et al. found that VitD could preserve the structure of elastic fibers and the ratio of elastic fibers to collagen in the tunica media of the aorta in experimental diabetes [69]. Since the increase in the collagen-to-elastin ratio could increase arterial stiffness [68], VitD appears to participate in the maintenance of the normal elasticity of the vessel wall.

**Figure 3:**
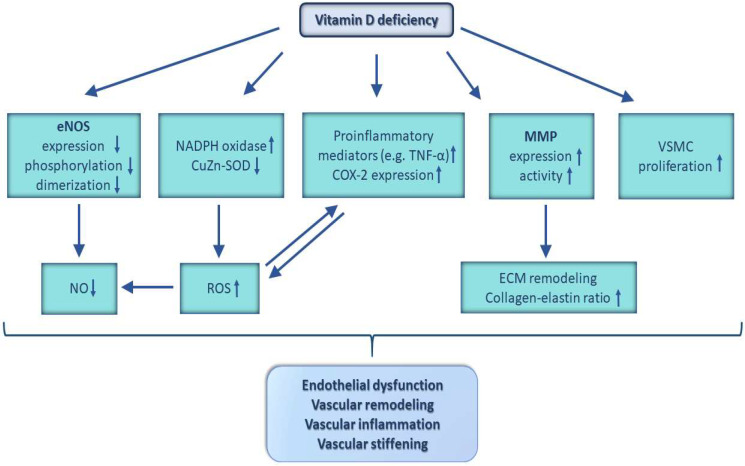
Representative actions of vitamin D deficiency in the (cerebro)vascular wall. Vitamin D deficiency impairs vascular functions via several pathways, including reductions in nitric oxide (NO) production, due to decreased endothelial nitric oxide synthase (eNOS) expression, phosphorylation, and dimerization. In addition, enhanced level of reactive oxygen species (ROS), resulting mainly from decreased expression of cytosolic copper-zinc superoxide dismutase (CuZn-SOD) and upregulation of the free radical generating nicotinamide adenine dinucleotide phosphate (NADPH) oxidase enzyme and its subunits in endothelial cells, contributes to endothelial dysfunction. Furthermore, vitamin D deficiency is associated with increased production of pro-inflammatory mediators, for instance, tumor necrosis factor-α (TNF-α) and cyclooxygenase 2 (COX-2), leading to vascular inflammation. Remodeling of extracellular matrix (ECM) and increased collagen–elastin ratio of vessel wall—particularly due to altered expression and activity of specific matrix metalloproteinases (MMPs)—as well as changes in vascular smooth muscle cell (VSMC) proliferation, may result in vascular remodeling and stiffening.

In summary, VitD is likely to be involved in the modulation of vascular remodeling, arterial stiffness, and angiogenesis. For instance, VitD has been reported to participate in the regulation of endothelial and smooth muscle cell proliferation, the extracellular matrix homeostasis, and the collagen–elastin content of arterial wall. Figure 3 represents various actions of VDD on the vascular wall, which may be responsible for vascular remodeling in VDD.

### 5.4. Impact of Vitamin D on Endothelial Function

#### 5.4.1. Vitamin D and the Nitric Oxide System

Normal endothelium-dependent, NO-mediated vasodilation is essential for maintenance of cardiovascular and cerebrovascular health [15,70,71,72,73,74]. Low levels of VitD are associated with diminished flow-mediated vasodilation in humans, which could be attributed to the endothelial dysfunction characteristic for VDD [32], as summarized in Figure 3. There is strong evidence that endothelial VDR plays an important role in preserving endothelial function [75]. According to in vitro studies using rodent and human tissues, VitD has been reported to upregulate the expression of endothelial nitric oxide synthase (eNOS) [76], to increase the dimer to monomer ratio of the eNOS protein [77], and to modulate the phosphorylation of eNOS [55,78], leading to increased eNOS activity and, thus, to enhanced NO production [79]. The vasoprotective action of VitD may involve activation of synergistic signaling pathways, including those mediated by cyclic adenosine monophosphate-activated protein kinase (AMPK) [55], the phosphoinositide-3-kinase (PI3K)/Akt, p38 mitogen-activated protein kinase (MAPK), and the extracellular signal-regulated kinase (ERK)/MAPK pathways [78]. In preclinical models, VitD treatment increased eNOS protein expression, improving endothelium-dependent vasorelaxation [80,81]. Conversely, in VDR-deficient mice, endothelium-mediated vasorelaxation of the aorta is impaired, due to reduced eNOS expression [75]. Additionally, diminished NO production has been reported in mice with functionally inactive VDR [37] and endothelial dysfunction in resistance arteries of rats exposed to early-life VDD [82]. In conclusion, according to human studies and animal experimentations, VitD may play an important role in preserving endothelial function, therefore preventing the development of age-related cardiovascular diseases.

#### 5.4.2. Oxidative Stress, Inflammation, and Vitamin D

VDD also promotes oxidative stress and inflammation, which may contribute to the genesis of both endothelial dysfunction and atherogenesis (Figure 3) [16,83,84]. For instance, VitD-deficient diet has been reported to increase the production of reactive oxygen species (ROS) in the aortic wall of rats [85] and down-regulate the antioxidant, ROS-scavenging enzyme, and cytosolic copper-zinc superoxide dismutase (CuZn-SOD) in the mesenteric arteries of mice [86]. VitD was also shown to decrease the expression of the ROS-generating NADPH oxidase enzymes in endothelial cells cultured from rodent and human tissues [77,84,87,88]. Thus, the anti-oxidative, vasoprotective action of physiological VitD levels is likely mediated synergistically by the upregulation of anti-oxidative pathways and down-regulation of pro-oxidative mechanisms [64,87]. By altering these pathways VDD may promote increased ROS production in the cardiovascular system [84]. The increase of oxidative stress, in turn, could lead to the inactivation of NO or oxidation of tetrahydrobiopterin, a critical cofactor for eNOS, which leads to eNOS uncoupling and, thus, to endothelial dysfunction [89].

VitD appears to suppress inflammation via several mechanisms, such as inhibition of the prostaglandin and cyclooxygenase pathways, upregulation of anti-inflammatory cytokines (interleukin (IL)-4 and IL-10), downregulation of pro-inflammatory cytokines (IL-1, IL-2, IL-6, IL-23, tumor necrosis factor-α (TNF-α), and interferon-γ), decreasing cytokine-induced expression of adhesion molecules, and downregulation of the RAS [32] [1]. In VDR-KO mice, the lack of VitD-mediated renin suppression leads to an increase in angiotensin II levels [52], which can promote vascular inflammation [83]. VitD has been reported to suppress the expression of inflammatory mediators, such as TNF-α, cyclooxygenase 2 (COX-2), and monocyte chemoattractant protein-1 (MCP-1), in the aorta of apolipoprotein E (ApoE)-deficient atherosclerotic mice [90]. Additionally, VitD downregulated the expression of COX-2 and the thromboxane prostanoid (TP) receptor in renal artery segments and aortic endothelial cells of ovariectomized rats, thus improving endothelial function [91]. Furthermore, VitD inhibits the activation of nuclear factor-κB (NF-κB) [92], and it decreases the expression of IL-6 in human endothelial cells [93]; thus, it prevents endothelial inflammation, improves flow-mediated vasodilation, and protects against atherosclerosis [1]. Correspondently, VDD may be associated with an increased production of pro-inflammatory mediators in mice [86], which is likely to promote vascular inflammation (Figure 3) [46]. In addition, animal models and clinical studies evidence that VitD suppresses the responses of T helper type 1 (T_H_1) and T helper type 17 (T_H_17) cells, whereas it supports regulatory T (T_reg_) and T helper type 2 (T_H_2) cells, which also contributes to the prevention of atherosclerosis [1,94,95].

The influence of VitD status on oxidative and inflammatory pathways suggests that VitD plays an important role in preventing oxidative stress and inflammation in the vascular wall. Thus, VDD is likely to increase the risk of vascular inflammation, contributing to the development of cardiovascular and cerebrovascular diseases.

### 5.5. Effects of Vitamin D on the Vascular Tone

As VitD influences endothelial function, especially NO bioavailability [1], it is not surprising that VitD contributes to the regulation of vascular tone. For instance, enhanced myogenic tone [82] and increased angiotensin II-induced vasoconstriction [86] of mesenteric arteries have been reported in the rodent models of VDD. Interestingly, in the coronary arteries of female rats, VDD appears to reduce both the pressure-induced myogenic tone and vasoconstriction evoked by a thromboxane A_2_ (TXA_2_) agonist [56]. In addition to the modulation of NO production, VitD appears to reduce the endothelium-dependent contraction of the aorta of spontaneously hypertensive rats, due to reducing calcium influx into the endothelial cells, thereby decreasing the production of endothelium-derived contracting factors [96,97]. Furthermore, VitD normalized the vascular reactivity of mesenteric arteries of spontaneously hypertensive rats via restoring the function of apamin- and ATP-sensitive K^+^ channels in VSMCs [98]. In conclusion, VDD might modulate vascular tone in various vascular beds, especially due to altering endothelial release of vasoactive mediators and reactivity to vasoconstrictor agents.

### 5.6. Impacts of Vitamin D on Vascular Permeability

VitD might play a role in preserving endothelial barrier function in various organs, including the brain, kidneys, and lungs [99,100,101,102,103]. In a combined rat model of chronic kidney disease and VDD, supplementation with paricalcitol (an active VitD analogue) resulted in the restoration of the impaired aortic endothelial permeability induced by chronic kidney disease [103]. Paricalcitol has also been reported to alter cell adhesion molecule expression and decrease vascular permeability in mice with endotoxemia [100]. In lungs, VitD might decrease alveolar–capillary permeability. In VDR-KO mice, increased permeability of alveolar wall has been reported, which was associated with altered expression of tight junction and adherens junction components [103]. According to a human study, high-dose treatment with oral cholecalciferol reduced the alterations in the pulmonary vascular permeability index in adults; however, the extravascular lung water index was not affected [101]. In the cerebrovascular system, VDD compromised the blood–brain barrier in a rat model of transient middle cerebral artery occlusion characterized by increased immunglobulin extravasation and decreased expression of tight junction proteins [102]. Thus, VDD may exacerbate the consequences of ischemic stroke partly due to increased blood–brain barrier permeability [102]. Furthermore, in a mouse model of cerebral cavernous malformation disease, cholecalciferol supplementation has been reported to reduce the lesion burden [104]. Additionally, cholecalciferol and its metabolites, 25(OH)D and 1,25(OH)2D, modulated endothelial stability via non-genomic actions; thus, they reduced vascular permeability in human endothelial cell monolayer and in mouse cerebral arteries [105].

## 6. Vitamin D Deficiency, Cardiovascular Diseases, Coronary Artery Disease, and Heart Failure

Cardiovascular diseases belong to the leading causes of morbidity and mortality worldwide [12,18]. The association between VitD levels and cardiovascular disorders has been widely investigated and extensively reviewed in the past few decades [1,12,18,34,106,107]. According to human studies, VDD has been linked to the increased risk of coronary artery disease (CAD), myocardial infarction, and heart failure [18,108]. However, whether VitD supplements exert a benefical effect on the development and progression of cardiovascular diseases is still under debate [12].

Since VDD is associated with several risk factors for cardiovascular diseases, it is likely that it increases the incidence of cardiovascular disorders indirectly. For instance, low VitD levels have been reported to increase the risk of hypertension, atherosclerosis, inflammation, and diabetes mellitus [106]. Of note, the causal associaton between some risk factors, for instance hypertension, and VDD are still not proven unambiguously [49]. VitD also might impact the pathogenesis of cardiovascular diseases, including CAD directly. In rodent models of VDD, morphological remodeling and altered vasoreactivity of intramural coronary arteries have been reported [109,110,111]. The aforementioned alterations in the coronary arterioles may impair the coronary circulation, therefore increasing the risk for cardiovascular disease. Endothelial dysfunction and subsequent atherosclerosis of coronary arteries are one of the leading causes for CAD [106]. As VitD improves endothelial function, due to several mechanisms, including preservation of endothelial NO and preventing vascular inflammation (see details above), it is presumable that VDD might contribute to the pathogenesis of CAD via facilitating endothelial dysfunction. Additionally, in VDD, the cholesterol uptake by macrophages is promoted, leading to increased macrophage-derived foam cell formation in the endothelium, which facilitates the progression of atherosclerosis [106]. In addition to the effects of VitD on coronary arteries, VitD has been reported to act directly on cardiomyocytes [22,40]. In VDR-KO mice, cardiac hypertrophy has been observed [29], whereas VitD treatment improved cardiomyocyte relaxation and, therefore, coronary perfusion during diastole in rodents [23]. Furthermore, VitD has been reported to play a role in extracellular matrix homeostasis in cardiomyocytes (see details above) [1]; thus, VitD influences heart failure development [12,112].

Taken together, VDD may favor the development of cardiovascular diseases, including CAD and heart failure, via influencing both coronary perfusion and cardiac function. Additionally, VDD has been linked to several risk factors of cardiovascular diseases; therefore, it also increases the incidence of CAD and heart failure indirectly. Table 2 summarizes the role of VDD in the pathogenesis of cardiovascular and cerebrovascular diseases.

## 7. Vitamin D Deficiency, Cerebrovascular Diseases, Stroke, and Vascular Cognitive Impairment

### 7.1. Role of VDD in the Pathogenesis of Cerebrovascular Disease and Stroke

Atherosclerotic cerebrovascular diseases and consequential ischemic strokes belong to the leading causes of death and disability worldwide [113,114,115]. Strokes due to atherosclerosis of a larger artery account for approximately one third of all stroke cases [116]. For instance, carotid artery atherosclerosis [117,118,119] may lead to ischemia, as a result of distal embolization or due to the hypoperfusion of brain tissue supplied by a severely narrowed or occluded vessel [116]. Strong epidemiological evidence link VDD to the increased risk of cerebrovascular diseases, including ischemic stroke [13,120,121,122,123,124]. VDD is particularly frequent in people who have suffered stroke, which is attributed to their limited mobility, advanced age, or malnutrition (i.e., conditions leading to decreased bioavailability of VitD) [13]. In humans, VDD has also been causally linked to poor post-stroke outcome [125,126], for instance, more severe cognitive impairment [13], and it is also associated with higher risk of death at one or two years following stroke and with greater risk of early recurrent stroke [13]. Although VDD appears to increase the risk for cerebrovascular diseases [13], large Mendelian randomization studies have failed to provide evidence for causal association between 25(OH)D levels and ischemic stroke [127,128,129]. Thus, furher studies are needed to establish the beneficial effect of VitD supplementation on reducing the incidence and severity of stroke [13,120]. Preclinical studies investigating the role of VitD in the outcome of stroke also yielded controversial results. For instance, in rodent models, VDD has been reported to increase the infarction volume, exacerbate the behavioral impairment, and compromise the blood–brain barrier after cerebrovascular occlusion [102,130]. Furthermore, VitD supplementation reduced the ischemia-induced brain damage in rodent brains [131,132]. Yet, in other rodent studies, VDD did not significantly affect the extent of brain injury following ischemic stroke [133]. Thus, further experimental studies using innovative models are warranted to characterize the impact of VDD on the cerebrovascular circulation. Epidemiological evidence also links VDD to chronic brain injury associated with cerebral small vessel disease [134], cerebral cavernous malformation disease [13], and increased arterial stiffness-related cerebrovascular damage [135].

Taken together, there is strong evidence, based on epidemiological and preclinical studies, that VDD is associated with cerebrovascular diseases, including ischemic stroke. Nevertheless, further studies are needed to establish the molecular and cellular mechanisms of the beneficial effect of VitD supplementation on reducing the risk and severity of ischemic stroke and to gain further insight into the cerebrovascular actions of VitD.

### 7.2. Role of VDD in the Pathogenesis of Vascular Cognitive Impairment

In addition to stroke, cerebrovascular diseases also manifest with cognitive impairment. The term vascular cognitive impairment (VCI) refers to diverse forms of cognitive disorders (ranging from mild cognitive impairment to vascular dementia), which associate with various cerebrovascular pathologies, ranging from cerebral small vessel disease [136,137,138,139,140,141,142,143,144,145,146,147,148,149,150,151] to large vessel atherosclerosis and stroke [152,153,154,155]. VCI is the second most common cause of age-related cognitive impairment and dementia after Alzheimer’s disease (AD). Additionally, the pathogenesis of AD itself also involves microvascular mechanisms; therefore, it is also considered a special form of VCI by many investigators [156,157,158,159,160,161,162,163,164,165]. In recent years, increasing evidence has been causally linked VDD to the genesis of VCI [166,167] and AD [168,169,170,171,172,173,174,175,176,177] in humans. Epidemiological studies suggest a more than doubled risk of mild cognitive impairment in older adults with VDD [178,179,180,181,182]. According to human studies, VitD levels also predict structural brain alterations, reduced hippocampal volume, and disrupted connectivity [183,184]. Further, VDR gene polymorphisms are associated with cognitive decline [185,186]. In conclusion, despite epidemiological studies implying a clear association between VDD and VCI, the underlying mechanisms are yet to be investigated.

Importantly, VCI and AD are quintessential diseases of aging. Their incidence exponentially increases with advanced aging, and the fundamental cellular and molecular mechanisms of aging contribute to their pathogenesis [187,188]. Importantly, there is initial evidence that VitD contributes to the regulation of epigenetic mechanisms of aging and that VitD supplementation decelerates epigenetic aging in subjects with VDD [189,190]. Other mechanisms of aging that may be affected by VDD include sirtuin pathways [191] and cellular senescence [192]. There is also evidence supporting an association between VDD and frailty in aging [193]. 

### 7.3. Effects of VitD and VDD on Cerebrovascular Homeostasis

VitD is known to influence several physiological processes relevant to cerebrovascular and brain pathophysiology [1]. Thus, VDD may affect cerebrovascular regulation and brain homeostasis and increase the risk and worsen the severity of ischemic stroke and VCI via multiple direct and indirect mechanisms. 

The direct effects of VDD by which it may increase risk of stroke include enhanced platelet aggregation, upregulation of tissue factor expression, downregulation of antithrombin and thrombomodulin expression, impaired biosynthesis of neurotrophic factors and neurotransmitters, and compromised detoxification pathways of the brain [120]. Since VDD is associated with several risk factors for stroke (Table 2), it may also increase the incidence of cerebrovascular diseases indirectly [120]. For instance, VDD is linked to hypertension [1], which is ultimately one of the major modifiable risk factors for cerebral ischemia [115]. Diabetes mellitus and insulin resistance are also associated with VDD, which may be attributed to the impaired β-cell function and insulin sensitivity of the target cells in VDD [194,195]. VDD stimulates the secretion of the parathyroid hormone; thus, it results in secondary hyperparathyroidism [196]. Since elevated parathyroid hormone levels have been found in stroke patients, an association between parathyroid hormone (PTH) levels and cerebrovascular diseases is presumable [196]. Furthermore, VDD favors inflammation [1], which may play a central role in the pathogenesis and progression of stroke [115]. In addition, VDD has been associated with subclinical carotid atherosclerosis [197]. Since atherosclerosis, particularly that of the carotid arteries, may lead to cerebral ischemia [196], VitD is likely to prevent stroke events, partly by being protective against atherosclerosis [16].

In order to provide continuous oxygen and nutrient supply for neurons, the cerebral circulation is tightly controlled by myogenic, metabolic, endothelial, and neurovascular mechanisms [73,198,199,200,201,202,203,204,205,206,207,208,209]. VitD also affects many of these critical mechanisms involved in the regulation of cerebral blood flow. The autoregulation of cerebral blood flow [210,211,212,213] ensures that cerebral blood flow remains constant, despite fluctuations in arterial blood pressure within the range of 60–150 mmHg [198,214]. However, when blood pressure is not within the limits of autoregulation, there is a risk of brain injury [215,216], including that of cerebral microhemorrhages [217,218]. The available preclinical evidence suggest that VDD may interfere with autoregulation of cerebral blood flow [219,220]. This concept is also supported by the findings that VDD impairs both cerebrovascular morphology and function in rodents [110,219,220,221]. Among others, VDD induces hypertrophic remodeling, resulting from enhanced vascular smooth muscle cell proliferation in the cerebral arteries of male rats [221]. VDD may also cause an increase in vessel tone and a decrease in endothelial relaxation capacity in the cerebral arteries of rats [221]. These alterations may result from enhanced vasoconstrictor prostanoid production, due to increased COX-2 expression and from decreased eNOS expression, leading to diminished NO production in cerebral vessels (Figure 3) [221,222,223,224]. Functional impairment and pathological remodeling of the cerebral vessels in VDD compromises cerebrovascular adaptation to ischemic events [110], increasing the risk for ischemic stroke and/or worsening its outcome [225,226,227,228,229,230]. Interestingly, in preclinical models, females appear to be better protected from the cerebrovascular effects of VDD than males; therefore, sex differences should be considered in clinical studies investigating the cerebrovascular manifestations of VDD, as well [219,220].

The critical neurovascular mechanisms by which functional and phenotypic alterations of the cerebral microcirculation [231,232,233] may promote the pathogenesis of VCI in older adults include cerebral microvascular rarefaction [234], impairment of neurovascular coupling responses (also known as functional hyperemia, which is responsible for moment-to-moment adjustment of cerebral blood flow to increased oxygen and nutrient demand of activated neurons [235,236,237,238,239,240]), and disruption of the blood–brain barrier [214,241,242,243,244,245,246,247,248]. VDR is known to be expressed on each cell type within the neurovascular unit, including endothelial cells, smooth muscle cells, pericytes [249], astrocytes, and neurons [250]. Thus, it is expected that VDD may affect the diverse physiological processes mediated by these cells. VDD was reported to aggravate capillary rarefaction in the kidney [251], whereas VitD was able to increase capillary density in the heart [252]. Yet, the effects of VitD and VDD on capillary density in the brain remains to be elucidated. There is promising preclinical evidence that VitD can improve blood–brain barrier integrity in various pathophysiological conditions [253,254,255]. Neurovascular coupling responses are, at least in part, mediated by endothelium-derived NO [256,257]. It is important, in that regard, that VDD was shown to improve endothelial function in vessels of the systemic circulation (see above). Thus, translational studies are warranted to explore the link among VDD, impaired neurovascular coupling responses, and impaired cognitive performance in older adults. The tone of cerebral resistance arterioles is also influenced by arterial partial pressure of carbon dioxide (pCO_2_) and, to a lesser extent, by partial pressure of oxygen (pO_2_) [198]. Importantly, hypoxia and hypercapnia promote the release of vasoactive mediators, including NO, from cerebral vessels, and the subsequent vasodilation increases the blood flow and, thus, tissue oxygenation [198]. It can be expected that changes in endothelial function due to VDD or VitD therapy would also affect the aforementioned vasoregulatory mechanisms. This hypothesis needs to be tested experimentally. Since the central nervous system is highly vulnerable to changes in cerebral blood flow [200], the synergistic impairment of multiple cerebral vascular homeostatic mechanisms is likely to exacerbate neurological disorders and promote cognitive decline [115,215]. 

## 8. Conclusions

Several human observational and animal experimental studies imply that VDD favors the development, worsens the outcome of cerebrovascular disorders, and promotes the genesis of cognitive decline. Therefore, the understanding of the mechanisms underlying the beneficial effect of VitD supplementation on reducing the incidence and severity of stroke, as well as the prevention of VCI, warrants further studies. Several questions remain to be answered. For instance, how do the time of onset and duration of VDD influence its cerebrovascular consequences? Are the cerebrovascular consequences of VDD reversible? Do cerebrovascular and cognitive outcomes correlate in clinical studies investigating the effects of VitD [258,259,260]? What is the mechanism underlying the sex-dependence of the cerebrovascular manifestation of VDD? How does VitD modulate the cellular mechanisms of aging in cells of the neurovascular unit? How do the effects of VitD on pathogenic mechanisms underlying VCI and AD differ [261,262]? How do combination treatments [263] consisting of VitD and specific interventions targeting cellular mechanisms of aging affect cerebrovascular and brain pathologies?

The high incidence of cardiovascular and cerebrovascular diseases worldwide, especially in advanced age, highlights the importance of investigating the roles and mechanisms of modifiable cardiovascular risk factors, including VDD, in the pathogenesis of vascular disorders. Although strong evidence links VDD to cerebrovascular diseases, the understanding of the complete mechanism underlying the impact of VDD on the development and severity of ischemic stroke and VCI warrants further well-designed experimental, epidemiological, and clinical studies. 

## Figures and Tables

**Table 1:** Selected VitD-regulated genes linked to cardiovascular and cerebrovascular functions.

Gene Name	Cell Type	Biological Function
Transforming growth factor, beta 2 and 3 (TGFB2, TGFB3) [33]	VSMC	cell proliferation
Prostaglandin-endoperoxide synthase 1 (cyclooxygenase 1) (PTGS1) [33,34]	VSMC, endothelial cell	prostanoid synthesis
Vascular endothelial growth factor (VEGF) [35,36]	VSMC, endothelial cell	angiogenesis
Tissue inhibitor of metalloproteinase 1 and 2 (TIMP1, TIMP2) [34]	cardiomyocyte	ECM homeostasis
Tissue inhibitor of metalloproteinase 3 (TIMP3) [35]	VSMC	ECM homeostasis
Matrix metalloproteinase 2 and 9(MMP2, MMP9) [34]	cardiomyocyte	ECM homeostasis
Nuclear factor kappa B (NFKB) [34]	endothelial cell	inflammation
Endothelial nitric oxide synthase (NOS3) [37]	endothelial cell	NO production
Interleukin 6 (IL6) [34]	endothelial cell	inflammation
Renin (REN) [38]	human embryonic kidney cells, mesangial cells	blood pressure, sodium retention

VSMC: vascular smooth muscle cell, ECM: extracellular matrix, NO: nitric oxide.

**Table 2:** Role of vitamin D deficiency (VDD) in the pathogenesis of cardiovascular and cerebrovascular diseases.

Risk Factor/Marker	Mechanism
Hypertension	Renin expression
Diabetes mellitus	β-cell function and insulin sensitivity
Cardiac hypertrophy	ECM remodeling
Atherosclerosis	Cholesterol uptake by macrophages
Vascular inflammation	Increased pro-inflammatory cytokines, decreased anti-inflammatory cytokines
Oxidative stress	ROS
Endothelial dysfunction	eNOS expression and activity, reaction of NO with ROS
Vascular permeability	Tight junction proteins
Vascular remodeling	VSMC proliferation, ECM remodeling, collagen–elastin content

ECM: extracellular matrix, ROS: reactive oxygen species, NO: nitric oxide, eNOS: endothelial nitric oxide synthase, VSMC: vascular smooth muscle cell.

## Data Availability

Not applicable.
